# The Intricate Balance between Life and Death: ROS, Cathepsins, and Their Interplay in Cell Death and Autophagy

**DOI:** 10.3390/ijms25074087

**Published:** 2024-04-06

**Authors:** Maya V. Voronina, Anastasia S. Frolova, Ekaterina P. Kolesova, Nikita A. Kuldyushev, Alessandro Parodi, Andrey A. Zamyatnin

**Affiliations:** 1 Research Center for Translational Medicine, Sirius University of Science and Technology, 354340 Sochi, Russia; voronina.mv@talantiuspeh.ru (M.V.V.); frolanasta@gmail.com (A.S.F.); kolesova.ep@talantiuspeh.ru (E.P.K.); kuldyushev.na@talantiuspeh.ru (N.A.K.); parodi.a@talantiuspeh.ru (A.P.); 2 Institute of Translational Medicine and Biotechnology, Sechenov First Moscow State Medical University, 119991 Moscow, Russia; 3 Faculty of Bioengineering and Bioinformatics, Lomonosov Moscow State University, 119234 Moscow, Russia; 4 Belozersky Institute of Physico-Chemical Biology, Lomonosov Moscow State University, 119992 Moscow, Russia; 5 Department of Biological Chemistry, Sechenov First Moscow State Medical University, 119991 Moscow, Russia

**Keywords:** oxidative stress, reactive oxygen species (ROS), cathepsins, cell death, apoptosis, autophagy

## Abstract

Cellular survival hinges on a delicate balance between accumulating damages and repair mechanisms. In this intricate equilibrium, oxidants, currently considered physiological molecules, can compromise vital cellular components, ultimately triggering cell death. On the other hand, cells possess countermeasures, such as autophagy, which degrades and recycles damaged molecules and organelles, restoring homeostasis. Lysosomes and their enzymatic arsenal, including cathepsins, play critical roles in this balance, influencing the cell’s fate toward either apoptosis and other mechanisms of regulated cell death or autophagy. However, the interplay between reactive oxygen species (ROS) and cathepsins in these life-or-death pathways transcends a simple cause-and-effect relationship. These elements directly and indirectly influence each other’s activities, creating a complex web of interactions. This review delves into the inner workings of regulated cell death and autophagy, highlighting the pivotal role of ROS and cathepsins in these pathways and their intricate interplay.

## 1. Introduction

While the intricate interplay between modulation of biochemical pathways and oxidative stress may not always be characterized by a straightforward cause-and-effect relationship, it undoubtedly represents a crucial juncture in cellular homeostasis and pathology. Many cellular processes are accompanied by the generation of reactive metabolites. Such metabolites include oxidants, which are generally defined as reactive oxygen (ROS), nitrogen (RNS), chlorine (RCS), and sulfur species (RSS). Oxidants can be interconverted into each other spontaneously or via catalytic aid [1,2]; for example, H_2_O_2_ (the most abundant physiological oxidant) can be converted into hydroxyl radical ^·^OH (the most potent physiological oxidant) [3] by interaction with appropriate electron donors, such as Fe^2+^, or into HOCl via myeloperoxidase activity in the phagosomes [4,5,6]. Major oxidants include hydroxyl radical (^·^OH), peroxynitrite (ONOO^−^), hypohalous acids (HOCl, HOBr, and HOSCN^−^), superoxide anion radical (O_2_^·−^), peroxides (ROOH) and peroxide radical (ROO^·^), reactive carbonyls (RC(O)^·^), and others [7]. Oxidant action depends on their activation energy, reaction kinetics, site of generation, and concentrations [2,8,9]. Oxidants have traditionally been viewed as harmful byproducts of cellular metabolic processes, such as the leakage of superoxide anion radicals (O_2_^·−^) from the mitochondrial electron transport chain into the cytosol [10] or during lipid metabolism via lipoxygenase activity, converting unsaturated fatty acids into oxidized products. However, more recently, it was shown that oxidant generation is tightly controlled [11] and has physiological functions, including cellular and intercellular signaling, fighting infections, and fine-tuned protein function regulation [12,13,14,15,16]. The proper regulation of these functions is determined by the cellular location of the enzymes that generate the ROS. In this scenario, the superoxide anion radical is “deliberately” generated together with H_2_O_2_ by NADPH-oxidizing NOX enzymes outside the mitochondria and by NADH-dependent enzymes inside the mitochondria [17]. H_2_O_2_ is recognized as a physiological second messenger, orchestrating many processes [7]. Due to its relatively low reactivity, H_2_O_2_ can travel long distances until it encounters an H_2_O_2_-sensitive site or is enzymatically scavenged [14,18]. Oxidative post-translational modifications in proteins can change the physical–chemical properties of amino acid residues, potentially leading to gain or loss of function and conformational changes [19,20,21]. Regulation of cellular and physiological processes is achieved by direct oxidation of sensitive sites or via targeted enzymatic oxidation of Cys and Met residues with thioredoxins, peroxiredoxins, and other enzymes, acting as redox switches [2,22,23]. In 2020, the visionary review of Lalmanach et al. [24] highlighted the key role of reactive oxygen species in regulating the activity of a particular class of proteases, referred to as cathepsins. Lysosomal cathepsins, a family of proteolytic enzymes mainly residing within the acidic lysosomal compartment, play a pivotal role in the regulation of the cellular processes, including (but not limited to) protein degradation, antigen presentation, and tissue remodeling [25,26], both in physiologic and pathologic conditions. Cathepsins are classified as a function of the presence of specific amino acids in their catalytic site in cysteine, serine, or aspartic proteases, or as a function of the substrate cleavage site in endo-, exo-, and endo/exopeptidase (Table 1).

The reciprocal regulation of ROS and cathepsins can potentially occur every time oxidative stress rises to a particular level of intensity, but the connection between these molecules is particularly evident in the cellular processes of controlled cell death (i.e., apoptosis, necroptosis, ferroptosis, pyroptosis, and NETosis) and autophagy, where ROS play a key role as inducers, while cathepsins represent the effectors of these phenomena. The interdependence of lysosomal cathepsins, oxidative stress, cell death, and autophagy underscores the multifaceted nature of cellular responses to environmental cues and stressors. Understanding the molecular intricacies of these interactions holds promise for unraveling novel therapeutic targets for conditions associated with dysregulated cell death and disrupted cellular quality control mechanisms. In this review, we describe the interactions between ROS and cathepsins, highlighting their reciprocal influence, in particular in apoptosis and autophagy. However, before describing the dynamics of this interplay in detail, some definitions of oxidative stress need to be reported.

## 2. Oxidative Stress General Definition and Methods of Investigation

**Figure 1:**
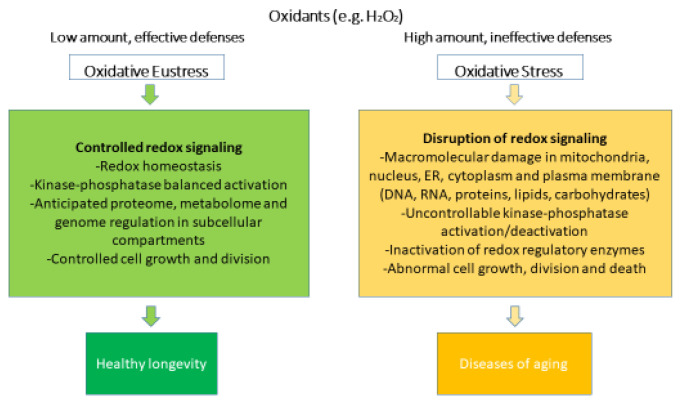
Oxidative stress and oxidative eustress within redox biology. Rearranged from [40].

Oxidative stress can be defined as the result of the disproportion between generated oxidants and antioxidants, which modulates or disrupts the redox signaling, leading to structural and functional molecular damages [39]. Currently, oxidative stress is classified into oxidative eustress and oxidative distress (Figure 1).

Oxidative eustress occurs after physical exercise [41] in response to small stressors, such as mild pharmacological interventions [42], and it is usually considered a physiological and endogenous process [43,44], also known as “mitohormesis” [44]. In oxidative eustress, low (nanomolar for H_2_O_2_) concentrations of oxidants interacting with their specific targets maintain the physiological signaling [45].

On the other hand, in oxidative distress, when concentrations of oxidants rise substantially (micromolar and millimolar for H_2_O_2_) [4], antioxidant systems are overwhelmed [46], leading to excessive oxidation of biomolecules. Reactive metabolites that are not scavenged by antioxidants may promiscuously react with proteins, DNA, and lipids. Oxidized molecules may show altered or loss of functions depending on the location of the oxidation site [47,48,49]. Oxidative distress is a hallmark of many pathologies, such as ischemia-reperfusion, sepsis, and aging [50].

To cope with the deleterious consequences of oxidative distress, several antioxidant and repairing systems have evolved [51,52,53,54,55,56,57]. Many of them rely on glutathione as a reducing equivalent. Glutathione is a short peptide used as a regenerative source for several antioxidant enzymes, such as peroxiredoxins, thioredoxins, and methionine sulfoxide reductases. Glutathione can also act alone, reducing oxidized Cys residues in a process known as glutathionilation [57]. Once oxidized, glutathione is reduced enzymatically by glutathione reductases, mostly in a NADPH-dependent manner [58]. Hydrogen sulfide H_2_S and persulfides (RSSH) may act as reductants, maintaining proper redox homeostasis and signaling [59,60,61]. Other small molecules, such as vitamins C and E, tocopherol, taurine, or protein-bound Met and Tyr, can directly scavenge the oxidants [52,53,55,62]. Monocytes secrete catalase, an enzyme that shields extracellular enzymes from oxidative damage [63]. Additionally, macrophages, when added to chemical and biological insults, can release cytotoxic ROS [64], and they can survive thanks to antioxidant enzymes, such as catalase [65]. 

There are several approaches to study oxidative stress in living (single) cells, dynamically and non-invasively, that implement more traditional tests based on chromatography and mass spectrometry that, on the other hand, require a relatively larger number of cells and their lysis. Small molecules, such as dichlorodihydrofluorescein diacetate, Amplex Red, or luminol, are convenient due to their ease of use and compatibility with primary cells, since they do not require any transfection or genetic manipulation. However, due to the absence of specificity and the potential generation of false-positive results, ROS identification with these dyes generally necessitates the use of orthogonal methods [66,67,68]. Recent genetically encoded redox indicators and sensors allow unambiguous detection of oxidants and oxidative stress effects with subcellular resolution. These molecular tools include sensors to measure markers of oxidative stress, such as a reduced/oxidized glutathione ratio [69], NADP+/NADPH [70], NAD+/NADH [71], redox potential [72], and Met oxidation [73,74,75], as well as sensors specific for oxidants, such as H_2_O_2_ [76], HOCl [77], and ONOO^−^ [78], and numerous sensors based on enzymes involved in oxidant-assisted signaling.

Both cathepsins and ROS can mutually influence each other’s activity and effects. For example, oxidative stress can influence the subcellular localization of cathepsins. Under normal conditions, cathepsins are predominantly localized within lysosomes, performing their proteolytic functions. However, oxidative stress can disrupt the integrity of lysosomes, causing their rupture and leading to the release of cathepsins into the cytosol, where they can contribute to pathological processes, such as apoptosis and inflammation [79].

On the other hand, the proteolytic activity of cathepsins can target the enzymes responsible for ROS generation [24]. More information about this phenomenon is provided in the text below.

## 3. Cathepsin and ROS in Apoptosis

Apoptosis is a vital cellular process, observed in all cell types, that involves distinct intrinsic and extrinsic pathways that converge on the activation of biochemical cascades, leading to cell death. Depending on the pathway, specific signals trigger mitochondrial membrane damage, resulting in the release of cytochrome C and ultimately leading to the activation of effector caspases. Effector caspases, along with mitochondrial proteins, translocate to the nucleus, where they carry out essential processes, such as nuclear protein cleavage, DNA fragmentation, and chromatin condensation. Apoptosis serves as a key mechanism in tissue maintenance, organ development, and immune system balance, ensuring cellular health [80]. However, apoptosis dysregulation can have significant implications for diseases, such as cancer, neurodegenerative disorders, and autoimmune conditions [81,82]. By targeting specific regulators and components of apoptotic pathways, it is possible to modulate cell death, offering potential therapeutic strategies for various disorders [83,84].

Emerging evidence suggests that cathepsin dysregulation can have significant implications in different cellular processes, including apoptosis [85]. In fact, while cathepsins may not play a predominant role in this process, they do contribute to apoptosis regulation by influencing some of its key steps. Cathepsins can act as regulators of apoptosis by affecting the activity of different enzymes. In the cytoplasm, cathepsins can directly cleave specific apoptotic regulators, such as pro-apoptotic and anti-apoptotic proteins. For example, research has shown that neutrophils isolated from cathepsin D-deficient mice undergo spontaneous apoptosis at later times compared to normal cells [86]. During this phenomenon, cathepsin D directly cleaves caspase-8, as demonstrated in both cellular and pure recombinant protein studies. Cathepsin D-mediated cleavage of caspase-8 produces an enzymatically active fragment, also known as initiator caspase, which further activates caspase-3 [86]. Cathepsin-mediated apoptosis can be induced by lysosomotropic agents, such as the 2-amino acid compound Leu-Leu-OMe, inducing the release of these enzymes in the cytoplasm. Upon this treatment, Bid cleavage and degradation of anti-apoptotic proteins, such as Bcl-2, Bcl-xL, Mcl-1, and XIAP, were detected in various cell lines [83]. Studies have shown that lysosomal proteases activate Bid protein in a time-dependent manner [87,88]. In this context, cathepsin B- and L-mediated activation of Bid and degradation of Mcl-1 were observed during Type-1-fimbriated *E. coli*-induced neutrophil apoptosis [42]. 

Oxidative stress plays a significant role not only in inducing, but also in regulating apoptosis [89,90]. As a trigger, ROS can induce various DNA and protein modifications, gene expression modulation, and increase mitochondrial membrane permeability [25]. If the damage inflicted by ROS becomes irreparable or overwhelms the cellular repair mechanisms, pro-apoptotic signaling pathways are activated [79,91]. For instance, mitochondrial membrane integrity disruption results in mitochondrial dysfunction and release of pro-apoptotic factors (i.e., cytochrome C). This, in turn, triggers the activation of caspases, culminating in apoptotic cell death [92]. ROS were particularly investigated as apoptotic inducers upon treatment with xenobiotics, such as cadmium, in osteosarcoma cells [93] or methacrylate monomers [94], which can induce apoptosis in dental pulp cells. In all these cases, antioxidant mechanisms or molecules were efficient in protecting the cells from apoptosis.

### Interplay between ROS and Cathepsins in Apoptosis

Oxidative stress can influence the subcellular localization of cathepsins by disrupting the integrity of lysosomes and causing their rupture, leading to the release of cathepsins into the cytosol, where they can contribute to apoptosis [87]. As a result of this phenomenon, recent studies have indicated that cathepsins can also translocate in the nucleus [95,96,97]. Interestingly, their activity within the nucleus appears to increase during apoptosis, even though more investigations are necessary to unveil the mechanisms underlying these phenomena [98]. Despite the role of ROS as a lysosome-permeabilizing agent, treatment of murine hepatoma cells with inhibitors of cathepsins L, B, and D did not prevent Bid activation after treatment with N-aspartyl chlorin e6 (NPe6) photosensitizer nanoparticles and subsequent irradiation [87]. In contrast to that described above, these data suggest that other lysosomal proteases might be involved in Bid activation under these specific conditions. On the other hand, a separate investigation observed changes in the expression and activity of cathepsins B and D in the rat pheochromocytoma cell line PC12 when treated with H_2_O_2_. Notably, H_2_O_2_ exposure increased cathepsin D activity, while cathepsin B activity remained unaffected [99]. During H_2_O_2_ and nitric oxide-induced apoptosis, cytoplasmic acidification is a well-established phenomenon. As cathepsins are known to activate at acidic pH within lysosomes, it was hypothesized that these proteases might also become active in the acidified cytoplasm during apoptosis. However, this hypothesis remains untested and requires further investigation [100].

The member of the Bcl-2 family Bax and its polyubiquitinated intermediate were found to be cleaved by cathepsin S during paclitaxel- or hydrogen-peroxide-induced apoptosis in renal cancer cells [101]. The activation of Bid and cleavage of anti-apoptotic proteins can result in mitochondrial outer membrane permeabilization and subsequent apoptotic stages, followed by caspase-3 activation. In this scenario, ROS, especially highly reactive hydroxyl radicals, can directly modify cathepsins’ activity. Specifically, Cys25, constituting the catalytic triad in the active center of cysteine cathepsins, can undergo post-translational modifications under the influence of ROS [24]. This modification can alter the active site of cysteine cathepsins and result in the inactivation of these enzymes [102].

However, moderate levels of oxidative stress can, in fact, activate pro-survival and pro-apoptotic signaling pathways simultaneously, allowing the cells to respond or to adapt to environmental conditions [103,104]. The precise outcome depends on factors such as the extent and duration of oxidative stress, the cellular antioxidant defense system, and the specific context and cell type involved. Cellular defense systems rely on antioxidant enzymes and molecules counteracting the effects of oxidative stress [25]. The disruption of molecular defense expression or function plays a crucial role in determining the fate of the cells, either promoting their survival or triggering apoptosis [105]. In this context, cathepsins have been implicated in regulating oxidative stress by controlling the expression and activity of antioxidant enzymes. A study investigating left ventricular (LV) dysfunction induced by overexpression of cathepsin A in cardiomyocytes revealed a reduction in the activity of the extracellular antioxidant enzyme, superoxide dismutase (EC-SOD), which catalyzes the dismutation of superoxide radicals to H_2_O_2_ and oxygen in the extracellular space. 

The decrease in EC-SOD in LV tissue in mice resulted in the accumulation of superoxide radicals that induced elevated expression of CTGF, TNF-α, IL-6, IL-10, and IL-2, and generated a high amount of apoptotic cells [106,107]. TNF-α is a cytokine produced by natural killer cells and cytotoxic T lymphocytes that induces various inflammatory and immune responses [108]. Furthermore, it was discovered that the inhibition of cathepsin B by Z-FA.FMK effectively blocked TNF-α/D-galactosamine-induced oxidative damage in the mouse brain. Injection of Z-FA.FMK resulted in decreased levels of lipid peroxidation and increased levels of glutathione. Additionally, the activity of catalase, superoxide dismutase, paraoxonase 1, and glutathione peroxidase increased compared with the TNF-α/D-galactosamine-treated group [109]. Cathepsin activity can also increase ROS production, via mitochondrial dysfunction. Conversely, in *S. cervi* parasites, inhibiting cathepsin D with E-64 caused a substantial reduction in glutathione levels, as well as glutathione reductase and glutathione-S-transferase activity, accompanied by an increase in NADPH oxidase activity. 

This resulted in an elevation of ROS, lipid, and protein peroxidation in the E-64-treated parasites [110]. Additionally, the inhibition of cathepsin K can disrupt the degradation of regulatory-associated protein of mammalian target of rapamycin (Raptor), resulting in heightened mitochondrial ROS levels [111]. To summarize, cathepsins have been found to regulate oxidative stress through their influence on antioxidant enzymes. In various systems, inhibiting specific cathepsins has shown promising results in mitigating oxidative damage and improving antioxidant defense mechanisms. 

The relationship between ROS and cathepsins is bidirectional: ROS can promote cathepsin release from lysosomes and affect the activity of cathepsin inhibitors. These interactions significantly impact the apoptotic pathway, ultimately influencing cell survival and death. Understanding the interplay between ROS and cathepsins in apoptosis can provide insights into the molecular mechanisms underlying cell fate determination and may have implications for developing therapeutic strategies targeting these pathways in various diseases.

**Figure 2:**
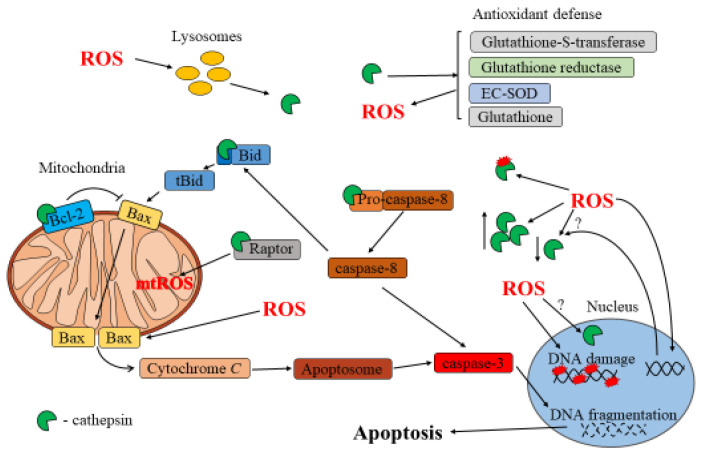
Interplay between ROS and cathepsins in apoptosis.

A schematic of the interplay between ROS and cathepsins during apoptosis is shown in Figure 2.

## 4. Other Types of Regulated Cell Death

The interplay between ROS and cathepsins was also highlighted in other forms of regulated cell death, including necroptosis, ferroptosis, pyroptosis, and NETosis.

Necroptosis is a form of programmed cell death resembling necrosis [112]. It was mainly observed during physiologic development or viral infection. Necroptosis pathways are usually associated with biochemical stimuli, including activation of death receptors (TNFR1 and Fas), toll-like receptors (TLR4), and others. When the necroptotic pathway is activated, receptor-interacting serine/threonine kinase 1 (RIPK1) associates with RIPK3 into a complex [113], which activates the mixed-lineage kinase domain-like pseudo-kinase (MLKL). This chain of events leads to post-translational modifications, resulting in the formation of a complex known as the necrosome (RIPK1–RIPK3–MLKL) [114]. The necrosome complex affects cell membrane continuity, eventually resulting in its permeabilization and cellular death.

Ferroptosis is a type of iron-dependent regulated cell death that is characterized by lipid peroxidation, leading to damage of the cell membrane [115]. The cell regulates the amount of iron through a system of transport, which involves transferrin and its receptor TFR for import and ferroportin for export. Cellular iron is transported as a complex with ferritin in its inactive form, Fe^3+^, predominantly localized in the cytoplasm, mitochondria, and nucleus [116]. The disruption of these iron transport systems and the release of iron from ferritin lead to the accumulation of intracellular iron, triggering ferroptosis. As a result of this process, lipid peroxidation occurs by iron-dependent enzymatic (lipoxygenases) and non-enzymatic (Fenton reactions) processes. These phenomena induce a significant oxidation of polyunsaturated fatty acids (PUFAs) in the cell membrane and organelles [117]. The glutathione peroxidase 4 (GPX4) complex plays a critical role in ferroptosis: its inactivation leads to the accumulation of PUFA-OOH [118]. Glutathione depletion, synthesized with the participation of system x_c_^−^ (SLC3A2 and SLC7A11) and used in GPX4 complex reactions, can trigger ferroptosis. Blockade of system x_c_^−^ triggers activation of voltage-dependent anion channel 2 (VDAC2) and VDAC3 on the outer mitochondrial membrane, leading to increased production of ROS [119]. The interplay between iron accumulation, glutathione depletion, ROS production, and increased lipid peroxidation ultimately drives ferroptotic cell death.

Pyroptosis is a type of regulated cell death observed during viral or bacterial infections, tissue damage, or metabolic disturbances, involving the activation of inflammasomes and consequent activation of pro-inflammatory caspases (such as caspase-1, 4, 5, and 11) [120]. These enzymes cleave interleukin-1 family members (i.e., pro-IL-1β and pro-IL-18) into their mature forms and gasdermin D (GSDMD) into two products: GSDMD-N and GSDMD-C. GSDMD-N can translocate to the inner layer of the cell membrane, binding cell phospholipids and affecting the continuity of this structure by generating pores. These pores allow the release of IL-1β and IL-18 from the cell, initiating an immune response. The damage to the cell membrane eventually leads to its rupture, which is a hallmark of pyroptosis.

Finally, NETosis is a specific form of cell death characteristic of neutrophils and other leukocytes (i.e., eosinophils, mast cells, and macrophages) [121,122,123] and is associated with release of neutrophil extracellular trap (NET) from the cell. Pathogens or external stimuli can trigger NADPH oxidase activation, inducing ROS production [124,125]. The azurophilic granules contain antimicrobial peptides, neutrophil elastase (NE), cathepsin G, and myeloperoxidase (MPO), which are released into the cell cytoplasm in response to ROS. NE moves into the nucleus, causing cleavage of nuclear proteins. Additionally, peptidyl arginine deiminase 4 (PAD4) leads to DNA de-condensation through histone citrullination. The decondensed chromatin, along with histones and proteases, is released into the cytoplasm and, subsequently, into the extracellular matrix, resulting in the formation of a neutrophil extracellular trap.

**Figure 3:**
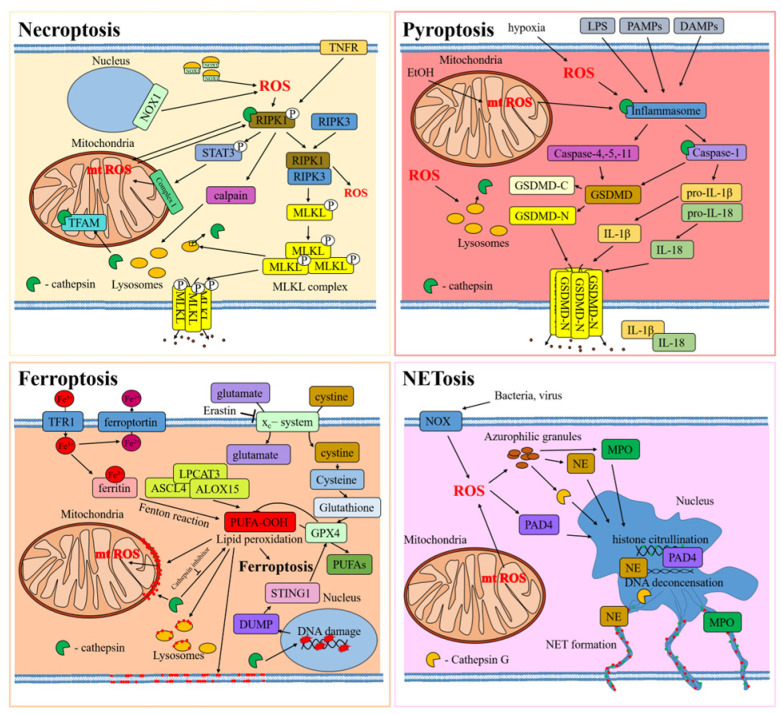
Interplay between ROS and cathepsins in types of regulated cell death. The cellular proteins are released from the cell through protein pores in the cell membrane, which are indicated by black dots. Red dots indicate lipid peroxidation in cell membranes.

In all these kinds of cellular death, the interplay between ROS and cathepsin is very similar (Figure 3).

ROS is a constant characteristic of necroptosis, ferroptosis, pyroptosis, and NETosis. In necroptosis, ROS can be produced through the activation of several pathways, including mitochondrial damage, that further increase ROS production [126]. For example, the transcriptional factor STAT3 is phosphorylated by the RIPK1 kinase, causing its translocation into the mitochondria, binding with respiratory chain complex I and increasing ROS levels [127]. The surge in mitochondrial ROS can, in turn, generate post-translational modifications in RIPK1, favoring the formation of the necrosome [128,129]. This phenomenon can further increase ROS levels [130], as observed in macrophages upon TNF-alpha activation [131]. Furthermore, in certain instances, ROS accumulation is directly linked to NADPH oxidase 1 activity, which can favor RIPK1 activation [132].

During ferroptosis, ROS and oxidation products can drastically increase, as observed during Fenton reactions, where H_2_O_2_ is converted to a hydroxyl radical (^·^OH). On the other hand, the enzymatic peroxidation of PUFAs, including phosphatidylethanolamine, occurs through a series of enzymatic reactions [133,134]. In these processes, iron ions play a key role as catalyzers. In this enzymatic balance, the GPX4 can mitigate ferroptosis activation and accumulation of oxidized PUFA. In this context, it was shown that erastin, a major activator of ferroptosis, can affect GPX4 activity by inhibiting glutathione formation, with consequent cell death by ferroptosis [119]. Similar to necroptosis, during ferroptosis, mitochondrial membrane undergo lipid oxidation, resulting in mitochondrial membrane damage [135,136] and a further increase in ROS production. 

Additionally, pyroptosis is associated with a significant increase in ROS levels that can eventually affect mitochondrial biology, fueling oxidative chain reactions. In cases of bacterial infection, cells exhibit increased production of ROS, resulting in lysosomal membrane permeabilization. 

In the process of NETosis, ROS are a key element in the formation of extracellular traps [137]. These ROS are essential in initiating the sequences that result in the release of NET. Studies have shown that the development of NET during fungal infection relies on NADPH oxidase, which generates ROS [138]. Additionally, ROS are responsible for triggering the release of azurophilic granules [139].

The oxidative stress generated during these kinds of cell death does not impact only mitochondrial biology but can also damage other organelles, including the lysosomes. An increase of ROS-mediated lysosome permeability was observed in necroptosis [140], ferroptosis [136,141], and pyroptosis during bacterial infection (Table 2) [142,143]. Cathepsin B was shown to play an important role in all these processes. In necroptosis, the necroptotic p-MLKL complex can be activated under ROS and is sequestered into lysosomes. This prompts the release of cathepsin B and further activates the pyroptotic cell death pathway [144]. In other works, together with cathepsin B, the release of cathepsins L and D was also shown [127,140,145,146,147,148,149]. The resultant release of lysosomal proteinases, including cathepsins, can further lead to the permeabilization of the mitochondrial membrane, increase of ROS, and ultimately, cell death [149]. In a different study, it was shown that the degradation of mitochondrial transcriptional factor A (TFAM) by lysosomal cathepsin B results in increased intracellular ROS levels that can eventually activate necroptosis [147], while in macrophages treated with LPS/zVAD, cathepsins B and L directly cleaved and activated RIPK1 [150].

During erastin-induced ferroptosis in PANC1 and MIAPaCa2 cells, lysosomes are destroyed and lysosomal proteases are released [141], and the activity and expression of cathepsins L and B were also found to be increased in glutamate-induced HT22 cells [136]. After being released into the cytoplasm of the cells, cathepsin B is transported to the nucleus, where it mediates DNA damage and releases nuclear DAMP into the cytoplasm. This, in turn, activates the STING1 pathway, leading to autophagy-dependent ferroptosis. The process results in the degradation of the antioxidant protein GPX4 and further ferroptosis [141]. In another study, inhibition of cathepsin B with CA-074-me reduced lipid oxidation, mitochondrial dysfunction, and ferroptotic cell death in spinal cord cells after spinal cord injury [151]. 

Following release from lysosomes during pyroptosis, which can be induced by ROS activity, cathepsin B activates formation of inflammasomes, such as NLRP3 [142,143,144]. Cathepsin B is necessary for inflammasome activation through its interaction with NLRP3 [152,153]. Experiments on mice that were fed either a special diet or acid showed an increase in the level of reactive oxygen species. This led to the release of cathepsin B, which activated the NLRP3 inflammasome [154]. 

Finally, during NETosis, cathepsin G, released from azurophilic granules, contributes to NET formation. Additionally, during this process, cathepsin G induces activation of cytokines IL-1α and IL-36 in PMA-treated neutrophils [155,156].

In conclusion, in these pathways, a vicious cycle between ROS and cathepsins occurs, eventually fueling cell death pathways.

**Table 2:** The relationship between cathepsin and reactive oxygen species in the pathways of various forms of cell death.

Cell Death	Cell Death Inducer	Cathepsin Assessment	ROS Assessment	Cells and Tissues	Trigger of the Interplay	Ref.
Necroptosis	Ischemic condition	Cathepsin release from lysosome undergoes lysosome membrane permeabilization.	Potential increase	Ischemic flaps	Cathepsins	[145]
Necroptosis	LPS + zVAD	CtsB and CtsL cleave RIPK1 protein. Cathepsin inhibition induces cell death.	Potential increase	Macrophages	Cathepsins	[150]
Necroptosis	Acute pancreatitis	Degrades TFAM.	Increase	Pancreatic acinar cells	Cathepsins	[147]
Necroptosis	TNF	Cathepsin L activation.	Increase	Mouse fibrosarcoma cells	ROS	[148]
Necroptosis	Tag7-Hsp70	Cathepsin B and D leakage from lysosomes.	Increase	Mouse fibroblast	Cathepsins	[149]
Necroptosis	FasL	Cathepsin B and D leakage from lysosomes.	Increase	Lymphoblast	Cathepsins	[127]
Necroptosis	Sodium sulfite	Cathepsin B and D leakage from lysosomes.	Increase	Mouse liver cells	ROS	[144]
Ferroptosis	Spinal cord injury	Increased Cathepsin B expression. CtsB inhibition decreases lipid peroxidation and mitochondrial disfunction.	Lipid peroxidation increase	Spinal cord	Cathepsins	[151]
Ferroptosis	Erastin	CtsB leakage from lysosomes. CtsB induces DNA damage.	Degradation of antioxidant protein GPX4	Pancreatic carcinoma cell	Cathepsins	[141]
Ferroptosis	Glutamate	CtsB is released from lysosomes, increases expression and activity, and cleaves H3.	Lipid peroxidation increase	Mouse hippocampal neuronal cell line	Cathepsins	[136]
Pyroptosis	*T. gondii* infection	CtsB release from lysosomes and its activation.	Increase	Human placental trophoblast, amniotic cells	Unknown	[142]
Pyroptosis	High-fat diet, palmitic acid	CtsB release from lysosomes and further NLRP3 activation.	Increase	C57BL/6J mice and AML12 cells	ROS	[154]
Pyroptosis	*B. cereus* strain, H2	Lysosomal damage and cathepsin release.	Increase	Macrophages	Unknown	[143]
Pyroptosis	All-trans retinal	Lysosomal damage and cathepsin release.	Increase	Spontaneously arising retinal pigment epithelia cells	Unknown	[153]
Pyroptosis	Sodium sulfite	Cathepsin release and NLRP3 activation.	Increase	Mouse liver cells	ROS	[144]
NETosis	PMA	Cathepsin contributes to NET formation.	Increase	Neutrophils	ROS	[139,156]

LPS: lipopolysaccharides; RIPK1: receptor-interacting serine/threonine kinase 1; CtsB: cathepsin B; CtsL: cathepsin L; FasL: Fas ligand; TFAM: mitochondrial transcription factor A; Hsp70: heat shock protein 70; ROS: reactive oxygen species; TNF: tumor necrosis factor; GPX4: glutathione peroxidase 4; NLRP3: NLR family pyrin domain containing 3; NET: neutrophil extracellular trap.

## 5. Cathepsin and ROS in Autophagy

Autophagy is the cellular process responsible for degrading and recycling cellular components through the formation of autophagosomes, playing a crucial role in maintaining cellular homeostasis and typical for eukaryotic cells [157,158].

**Figure 4:**
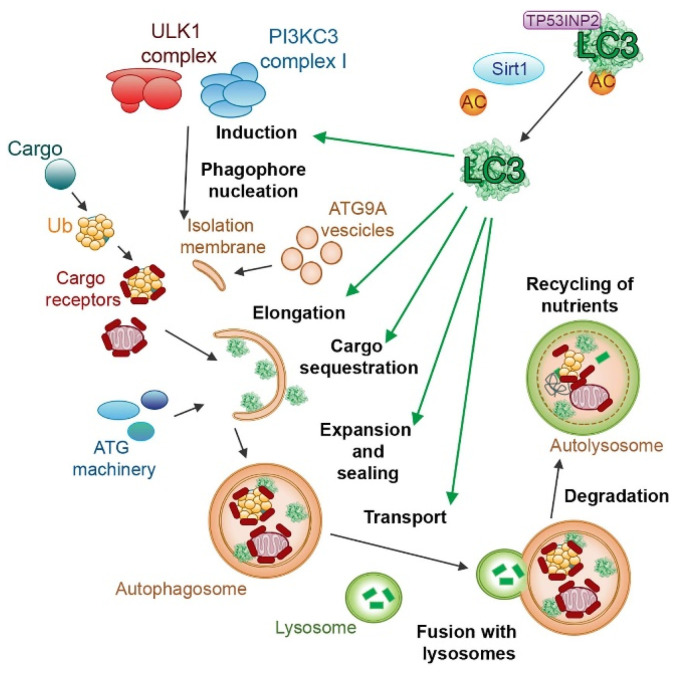
The role of LC3 proteins in selective autophagy. The figure illustrates the involvement of the LC3 subfamily of ATG8 proteins in different steps of selective autophagy. Reproduced with permission from [172].

This process can be stimulated by different kinds of cellular stress, such as starvation, hypoxia, oxidative stress, DNA damage, and intracellular pathogens [159,160,161], even though a certain degree of genetic correlation between autophagy and senescence was demonstrated [162]. Cellular senescence is another pathway of stress response [163,164,165], and this correlation has been the subject of numerous studies [166,167], demonstrating the activation of similar signaling pathways between these processes [168,169,170]. Recent studies have revealed a complex interplay between autophagy and oxidative stress, where autophagy serves as a key mechanism to mitigate cellular damage by selectively removing damaged organelles and protein aggregates generated under oxidative stress conditions [171]. In response to lipid and protein oxidation, the redox-regulated protease ATG4 cleaves Atg8/microtubule-associated protein light chain 3 (LC3), inducing its binding to phosphatidylethanolamine (PE) and its localization on the autophagosomal membrane (Figure 4).

ATG5, ATG7, and other modifiers also play a role in the maturation of autophagosomes and in the formation of autolysosomes.

Autophagy regulation is orchestrated by a complex interplay between three main signaling pathways. The class I phosphatidylinositol 3-kinase (PI3K) pathway is activated by growth factors, promoting cell growth, while the class III PI3K pathway responds to the number of amino acids within the cell. When amino acids are scarce, this pathway triggers autophagy to recycle cellular components for survival. Similarly, the LKB1/AMPK pathway primarily reacts to cellular ATP levels. When ATP is low, it activates autophagy to generate energy by breaking down cellular components. mTOR kinase acts as a central checkpoint for autophagy, functioning as its main repressor in these pathways when the cell has sufficient resources. Another key player in autophagy is the extracellular signal-regulated kinase (ERK), which can induce cytoplasm vacuolization [173] and induction of LC3, beclin-1, and p53 phosphorylation [174], all of which are involved in regulating this process.

Autophagy can be induced as a protective response against oxidative stress, promoting cell survival through the removal of dysfunctional mitochondria, which are major sources of ROS generation [175,176,177]. Autophagy dysregulation was linked to various diseases, including neurodegenerative disorders and cancer, emphasizing the need to elucidate the molecular mechanisms driving this process for effective therapeutic strategies and understanding pathologic mechanisms. Lysosomes play a pivotal role in autophagy, serving as the final destination for the degradation of sequestered cytoplasmic components delivered through autophagosomes [178,179]. In this scenario, cathepsins are the key mediators of the autophagic flux by cleaving and breaking down the cargo within lysosomes, ensuring the efficient degradation of proteins, organelles, and other cellular structures [180,181]. Stringent regulation of cathepsin expression and activity ensures efficient lysosomal degradation, preventing uncontrolled proteolysis and promoting controlled autophagic cargo breakdown. Cathepsins also contribute to autophagy modulation by participating in the regulation of autophagosome–lysosome fusion, a crucial factor that influences the efficiency of this process [182,183]. For example, a mutation in the lysosomal factor Saposin C, that favors the activity of acid β-gluocosidase, can induce aberrant autophagy by inducing accumulation of autophagosomes and decrease cathepsin B and D expression and activity [182]. Furthermore, studies have shown that inhibiting cathepsin S can directly induce autophagy. This activation occurs through the phosphorylation of the epidermal growth factor receptor (EGFR), leading to the activation of the ERK/MAPK signaling pathway, which is known to regulate autophagy [184].

### Interplay of ROS and Cathepsins in Autophagy

**Figure 5:**
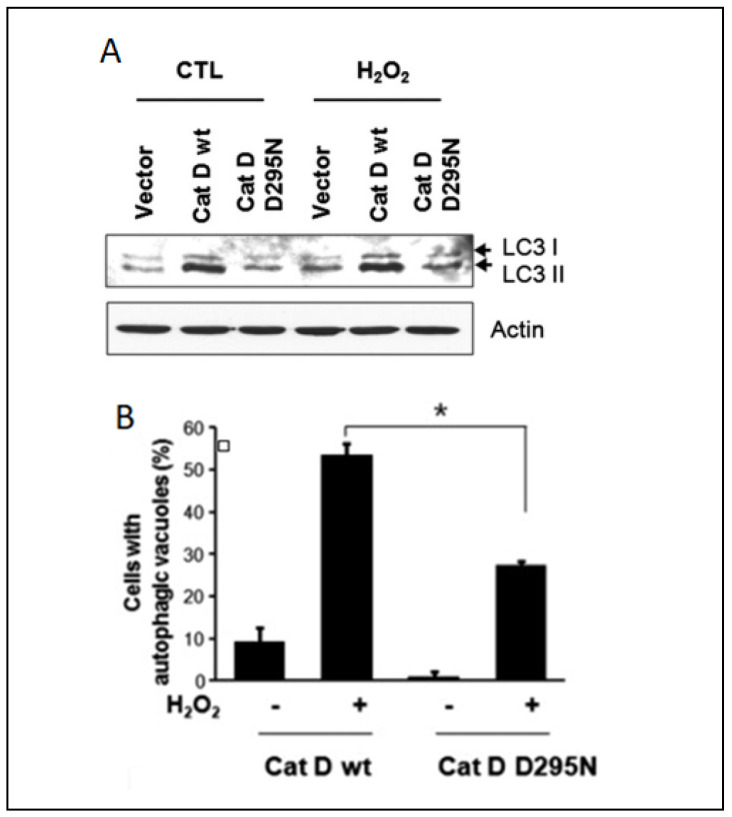
Correlation between cathepsin D expression and autophagy in HeLa cells: (**A**) Western blotting analysis of HeLa cells expressing wildtype cathepsin D and cathepsin D D295N after treatment with 1 mM of H_2_O_2_ for 24 h. (**B**) Percentage of HeLa cells with autophagy vacuoles after H_2_O_2_ treatment. Data were obtained from confocal images of cells transfected with GFP-LC3 plasmid. +/− means HeLa cells with or without H_2_O_2_ treatment, * indicates the significance. Reproduced with permission from [188].

Extensive research has investigated alterations in the proteolytic activity during autophagy, but the underlying mechanisms orchestrating the autophagic processes in response to oxidative stress remain elusive. On the other hand, substantial attention has been devoted to the intricate relationship between autophagy and apoptosis [185,186]. While autophagy typically exerts a protective and anti-apoptotic function, it can trigger programmed cell death under conditions of extreme external stress [187], and oxidative stress and cathepsins cover a key role in this fine balance. It was demonstrated that cathepsin D can enhance the survival of HeLa cells under oxidative conditions, challenging the established link between apoptosis and autophagy. The authors hypothesized that elevated cathepsin D expression activates autophagy, and this phenomenon was substantiated by increased efficiency in autophagic vacuole formation and the autophagy marker LC3-II (Figure 5).

**Figure 6:**
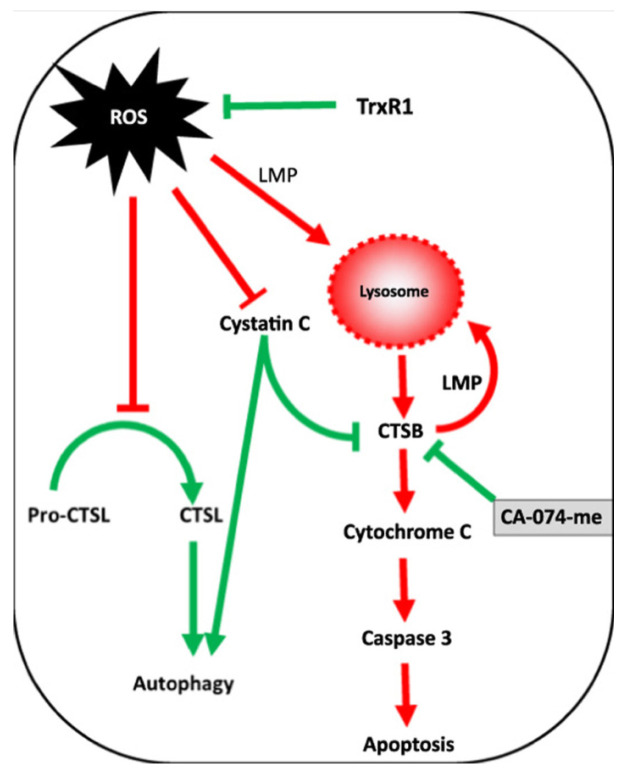
Correlation between ROS, cathepsin B (CTSB), and cathepsin L (CTSL) in regulating autophagy and apoptosis as a result of thioredoxin reductase inhibition. Reproduced with permission from [190].

Conversely to the studies reported in the apoptosis section, these findings indicate that cathepsin D can act as an anti-apoptotic mediator by inducing autophagy during cellular oxidative stress. A similar anti-apoptotic effect of cathepsin D was also observed in colorectal cancer cells [189]. Another investigation revealing a connection between oxidative stress and cathepsin L and B activity indicated these proteases as pro-autophagic and pro-apoptotic enzymes, respectively [190]. It was observed that oxidative stress induced by auranofin-mediated inhibition of thioredoxin reductase led to a significant increase in cathepsin B activity, while the protein levels of this enzyme remained relatively unchanged. Conversely, cathepsin L exhibited an opposite pattern, with a substantial increase in protein levels not accompanied by a corresponding change in activity. The authors demonstrated, via the thiol-trapping method, that the oxidative stress disrupts cathepsin L processing, impairing its pro-autophagic function. However, no discernible impact of oxidative stress on cathepsin B was identified. The authors proposed the following mechanism to account for these observations: protective autophagy prevents oxidative stress by inhibiting cathepsin L processing, while apoptosis is induced by an increased lysosomal membrane permeability that favors cathepsin B release into the cytoplasm, which can eventually induce the activation of pro-apoptotic enzymes. Inhibition of cathepsin B under these conditions suppresses apoptosis, enhancing cell viability. The interplay of this mechanism is shown in Figure 6. 

A recent study has shed light on the interplay between cathepsin S and autophagy. This study demonstrated that the autophagic process is accompanied by an increase in ROS; in fact, by inhibiting the expression of ATG-related proteins via gene silencing or pharmacological agents, ROS levels decreased. On the other hand, the inhibition of cathepsin S induced ROS production and autophagy, as well as DNA damage. The authors indicated that the enzyme xanthine oxidase is at the basis of the working mechanism regulating the balance between autophagy and cathepsin S activity. However, while xanthine oxidase is a key player in ROS generation during autophagy, direct proof of the interaction between this enzyme and cathepsin S was not reported. Additionally, the relationship between cathepsin E and oxidative stress during autophagy was analyzed in mice macrophages [191]. In this context, cathepsin E-deficient murine macrophages displayed an aberrant autophagic behavior, characterized by heightened levels of autophagy markers, such as LC3 and phosphorylated p62, which can cause autophagy [192]. Cathepsin E deficiency also induced perturbations in signaling pathways associated with autophagy, impacting mTOR and ERK signaling. Furthermore, cathepsin E deficiency hindered the fusion of autophagosomes with lysosomes via inhibition of LC3 transport to the vesicular compartment. The macrophages exhibited an increase in ROS levels accompanied by the activation of oxidized peroxiredoxin-6 and a concomitant reduction of glutathione. Hence, it can be postulated that cathepsin E can exert a substantial influence on oxidative stress, operating through a NADPH oxidase-independent pathway. The aforementioned studies collectively underscore a robust correlation between oxidative stress, autophagy, and lysosomal cathepsins. Targeting cathepsins could thus be contemplated as a viable strategy for the manipulation of autophagy, early ROS generation, and cell death, potentially offering therapeutic avenues for a spectrum of diseases.

## 6. Conclusions

ROS can have a destructive effect on cellular structures and initiate free radical oxidation of nucleic acids, lipids, and proteins, which underlie the pathogenesis of many diseases. These phenomena usually activate proteolytic processes that can determine cell life or death in the contexts of autophagy and cell death, respectively. Cathepsins can regulate these phenomena as effectors or indirectly, favoring the development of these processes. In this review, we highlighted different mechanisms in which cathepsins regulate apoptosis and other kinds of cell death and autophagy induced by ROS. However, the interplay between ROS and cathepsins is significantly more complex than a simple cause–effect relationship since they can directly and reciprocally affect each other’s function and activity. For this reason, further investigations are needed to understand the fine regulation of the proteolytic machinery during oxidative stress and the contribution of cathepsin in degrading antioxidant enzymes. These studies might reveal new targets for the treatment of different diseases since ROS is a common element in many pathological conditions. For this reason, parallel efforts should be performed in developing more sensors to precisely understand the oxidant origin, conversion, and effects on different molecules and, in particular, on cathepsins.

## Figures and Tables

**Table 1:** Cathepsin activity, expression, and gene name. Table implemented from [27].

Classes of Proteases	Cathepsins	Gene Name	Molecular Weight (Da)	Peptidase Activity	Expression
**Serine**	Cathepsin A	CTSA	~100,000–600,000	Carboxypeptidase	Lymphoblastoid cells, primary human B cells, both subsets of myeloid dendritic cells (mDC1 and mDC2), as well as in plasmacytoid DC [28]
	Cathepsin G	CTSG	~27,000–30,000	Endopeptidase	Neutrophil [29], human renal, and breast cancer cells [30]
**Cysteine**	Cathepsin B	CTSB	~25,000–29,000	Carboxydipeptidase, Endopeptidase	Ubiquitous
	Cathepsin C	CTSC	~200,000	Aminodipeptidase, Exopeptidase	Ubiquitous
	Cathepsin F	CTSF	~50,000–70,000	Endopeptidase	Ubiquitous
	Cathepsin H	CTSH	~28,000	Aminopeptidase, Endopeptidase	Ubiquitous
	Cathepsin K	CTSK	~650,000	Endopeptidase	Osteoclasts [31]
	Cathepsin L	CTSL	~24,000	Endopeptidase	Ubiquitous
	Cathepsin O	CTSO	~23,460	Endopeptidase	Ubiquitous
	Cathepsin S	CTSS	~14,000–17,000	Endopeptidase	Antigen-presenting cells [32,33]
	Cathepsin V	CTSV	~35,000	Endopeptidase	Thymus, testis [34,35]
	Cathepsin W	CTSW	~43,000	Endopeptidase	Natural killer cells, cytotoxic T cells [36]
	Cathepsin Z (Cathepsin X)	CTSZ	~53,000	Carboxymonopeptidase Exopeptidase	Ubiquitous
**Aspartyl**	Cathepsin D	CTSD	~42,000	Endopeptidase	In practically all tissues and organs [37]
	Cathepsin E	CTSE	~100,000	Endopeptidase	Pancreatic ductal adenocarcinoma [38]
